# Understanding human co-manipulation via motion and haptic information to enable future physical human-robotic collaborations

**DOI:** 10.3389/fnbot.2025.1480399

**Published:** 2025-06-19

**Authors:** Kody Shaw, John L. Salmon, Marc D. Killpack

**Affiliations:** Robotics and Dynamics Laboratory, Department of Mechanical Engineering, Brigham Young University, Provo, UT, United States

**Keywords:** co-manipulation, physical-interaction, multi-agent, haptics, human collaboration and interaction

## Abstract

Human teams intuitively and effectively collaborate to move large, heavy, or unwieldy objects. However, understanding of this interaction in literature is limited. This is especially problematic given our goal to enable human-robot teams to work together. Therefore, to better understand how human teams work together to eventually enable intuitive human-robot interaction, in this paper we examine four sub-components of collaborative manipulation (co-manipulation), using motion and haptics. We define co-manipulation as a group of two or more agents collaboratively moving an object. We present a study that uses a large object for co-manipulation as we vary the number of participants (two or three) and the roles of the participants (leaders or followers), and the degrees of freedom necessary to complete the defined motion for the object. In analyzing the results, we focus on four key components related to motion and haptics. Specifically, we first define and examine a static or rest state to demonstrate a method of detecting transitions between the static state and an active state, where one or more agents are moving toward an intended goal. Secondly, we analyze a variety of signals (e.g. force, acceleration, etc.) during movements in each of the six rigid-body degrees of freedom of the co-manipulated object. This data allows us to identify the best signals that correlate with the desired motion of the team. Third, we examine the completion percentage of each task. The completion percentage for each task can be used to determine which motion objectives can be communicated via haptic feedback. Finally, we define a metric to determine if participants divide two degree-of-freedom tasks into separate degrees of freedom or if they take the most direct path. These four components contribute to the necessary groundwork for advancing intuitive human-robot interaction.

## 1 Introduction

The capability for humans and robots to perform collaborative manipulation tasks has the potential to improve quality of life and provide assistance in difficult or time-sensitive circumstances (Goodrich and Schultz, 2008). Applications include search and rescue, disaster response and cleanup, construction, and logistics. Unfortunately, for reasons ranging from hardware limitations to safety and effective control, robots still require significant development in complex cooperative or co-manipulation tasks (Martinetti et al., 2021; Villani et al., 2018).

In this paper, we describe a multi-agent co-manipulation study and explore four fundamental categories of importance for future human-robot co-manipulation. These include the definition of a rest state, signals communicating motion primitives (i.e., small sub-movements that that can be integrated to result in larger complex motion), communicability of desired intent across degrees of freedom, and how people divide or combine degrees of freedom (DOF) in multi-DOF tasks. We define motion primitives as simple predefined movements in each of the 6 DOFs during co-manipulation. We define co-manipulation as the efforts of two or more individuals collaboratively moving an object. People have been working together in this way long before recorded history, hence it is completely natural for us to gather around an object too large for an average individual to manipulate alone, and lift and move it together. Humans rarely need precise visual or auditory instruction to follow a desired path successfully. How do we communicate our intent? What are the limits of our ability to intuit or communicate a path based on haptics? These are broad, overarching questions that we propose need to be answered to understand and enable future efforts in human-robot co-manipulation. Our specific interest is in co-manipulation of objects with significant mass or extent that would be difficult or unreasonable to expect an average person to move alone.

We will address four specific sub-components of these questions by analyzing how human-only teams behave, which can then be applied in future human-robot co-manipulation. First, we analyze the static state, which is defined as when a team or leader has no intentional movement. Identifying and quantifying important signals about this state will allow a robot to recognize when to wait, and when it is time to leave the static state. Secondly, once moving, an assessment of several commonly used signals is undertaken to determine which is best to identify a desired motion primitive or direction of motion. Third, for each DOF we explore how well a desired motion primitive can be communicated through haptic feedback alone. Finally, given a task requiring the motion of the co-manipulated object in two DOFs, we determine whether participants opt to handle one DOF at a time or accomplish both DOFs simultaneously.

In summary we contribute the following findings.

- A method of identifying the transition from the static to an active state.
- Quantifying the best signal to use in the prediction of future motion.
- An itemized list of which DOFs can and cannot be communicated through haptic feedback, as well as a first-order ranking of the difficulty of communicating each DOF.
- An analysis of the the paths taken when participants are required to perform tasks requiring 2 DOF.

For each of these contributions, we also explored whether any significant effects occurred due to three different leadership types which depend on what information is available to each participant: leader-leader teams (LL), leader-follower teams (LF), and a team with a leader and two followers (LFF). We define a leader as a participant who is given direct information about the task and therefore guides the overall motion to a final configuration. In LL, both participants are given the same instructions simultaneously, resulting in near-ideal coordination. In contrast, for LF and LFF, instructions were only provided to the leader. Furthermore, the leaders were not permitted to use verbal communication, and thus were restricted to only using the haptic communication channel, with signals, forces, and displacements expressed through the motion of the object. The LF type allows us to gain insights into the use and limitations of haptic communication which is the signal most readily available to future robotic teammates. Adding an additional follower in LFF also allows us to determine the effects of having two participants in the group who must receive the same message via haptic feedback.

## 2 Prior work

This section explores five key areas related to intuitive human-robot interaction development. We explore current works in the following areas activation thresholds, motion primitives, co-manipulation signals, leader follower relationships, and several similar human-human studies.

### 2.1 Activation thresholds

When operating with a team, knowing when to move and when to remain motionless is an important distinction for a robot to be able to make. If a robot is too sensitive, it will respond to unintended signals. On the other end of the spectrum, if a robot is not sensitive enough it will be sluggish and slow to respond. Dumora et al. (2012) explored this idea as they were exploring the translation vs rotation problem. They reported that forces over 5N for more than 100 ms or torque over 0.7 Nm for more than 100 ms, indicated an intentional act. We are exploring this claim in more detail by considering levels of variation or noise in signals such as position, velocity, and acceleration while participants are at rest in order to help determine appropriate levels of sensitivity.

### 2.2 Motion primitives

Motion primitives (or small sub-movements that can be combined to complete a larger, more complex movement goal) have been widely accepted to analyze human motions and improve robotic movement performance for some time (Hofsten, 1992; Stulp et al., 2012; Burdet and Milner, 1998; Milner and Ijaz, 1990). The idea of motion primitives has also been implemented into many robotic systems, with some success. These sub-motions, or motion primitives, have been applied to robotic manipulation in many areas not necessarily related to co-manipulation. For example, Pook and Ballard (1993) made use of motion primitives to flip an egg via a robotic arm. Bentivegna and Atkeson (2001) used motion primitives to simplify the learning process for a robot that played air hockey.

Specific to human-robot co-manipulation, Bussy et al. (2012), used ideas obtained from Corteville et al. (2007) and Maeda et al. (2001) on predicting human intentions via a simple set of motion primitives in planar 3-DOF co-manipulation of a wooden frame. They predefined these motion primitives as, forward, stop, turn, and sideways. By using this approach, they were able to create a proactive follower that guessed the desired sub-motion, which, they reported, substantially reduced the “workless interaction force” (Bussy et al., 2012), or unnecessary planar load. Lanini et al. (2018) used a similar approach using start and stop motion primitives to co-manipulate a pole while estimating a desired velocity along a single axis. It has been hypothesized that the benefits of motion primitives can be extended by increasing their number and scope. However, increasing the number of motion primitives also increases the difficulty of determining which motion primitives are desired by a group. This paper begins the process of extending motion primitives from start, stop, turn, etc., to a more complete set with a motion primitive in each DOF. The motion primitives are aligned with the DOF of an inertial frame which is aligned with the frame of the object in its starting position. In this paper, we examine several signals to determine whether they can be used to consistently predict motion primitives in each DOF.

### 2.3 Signal space

A wide variety of signals have been used to try and understand co-manipulation and enable robots to effectively assist humans. For example, work by Dumora et al. (2013) demonstrated that robots can dynamically adjust their assistance strategies based on real-time haptic feedback. Similarly, other papers cite the use of haptic feedback as a beneficial method to collaborate with robots (see Agravante et al., 2013; Mörtl et al., 2012; Van Der Wel et al., 2011; Takagi et al., 2019, 2018; Dumora et al., 2012; Madan et al., 2015). Robotic vision is another channel that has been used to try and improve performance in human-robot co-manipulation tasks (Yu et al., 2021; Agravante et al., 2013; Deegan et al., 2022; Abu Bakar et al., 2010; Groten et al., 2009). Even the auditory channel has received some attention (Jensen et al., 2021; Ibarguren et al., 2020; Griffin et al., 2005; Bakar et al., 2007; Ghadirzadeh et al., 2016). A few papers have considered the relative value or quality of particular signals. For example, Ibarguren et al. (2020) reported that 38% of their participants preferred audio. Abu Bakar et al. (2010) reported that vision and touch were the most used signals and suggested that knowledge of the goal could also greatly improve performance. Mojtahedi et al. (2017) showed that the reduction of impedance in the desired direction can be used to infer the intended movement. Bin Abu Bakar et al. (2006) reported in their human-human study, that displacement torque had little bearing on a follower's ability to be a proactive assistant, but that velocities of sufficient magnitude were critical. Ikeura and Inooka (1995) also found that human followers used the movement of the object to determine when to actively assist. We take a more in-depth look into acceleration, velocity, and several different measures of force that can be readily measured by a robot, and determine how they might be used to improve human-robot co-manipulation.

Jensen et al. (2021) also claimed “haptic feedback alone represents a sufficient communication channel for co-manipulation tasks”. In this work, we explore this claim in more detail. We aim to answer questions such as which co-manipulation tasks can be communicated over haptics, and how performance is affected when communication is restricted to the haptic channel.

### 2.4 Leader-follower relationships

It is important to first define what we mean in this paper as definitions for leaders and followers. We follow the definitions from human-human co-manipulation studies like Ikeura and Inooka (1995), Abu Bakar et al. (2010), and Mielke et al. (2017) where the leader is expected to determine the overall motion of the object because of information they have about the goal and objective, and the follower is expected to be supportive (i.e. not antagonistic) in achieving the task. This also mirrors a number of human-robot studies where the human is intended to explicitly be the leader and generate the motion for a robot to follow (see Arai et al., 2000; Dumora et al., 2012). We expect that this definition may be the most useful as we analyze human-human data in this paper with the objective of applying the results to human-robot systems with the human as the assumed leader initially. However, there are a wide variety of definitions of leader-follower teams in the literature that range from a definition based on relative stiffness (Vianello et al., 2022) to roles that adapt or change over the course of the task (Nakayama et al., 2017).

More specific to our analysis of the effect of LL and LF teams on task performance in this paper, some researchers have begun to also explore LL and LF leadership types. For example, Agravante et al. (2016) and Whitsell and Artemiadis (2017) examine how roles change or are exchanged during co-manipulation tasks. In a two-person box lifting experiment, Abu Bakar et al. (2010) reported that the smoothness of a task improved if the follower knew the start and end locations of the goal. In this work, we expand on Bakar's work by comparing three leadership types—LL, LF, and LFF. We aim to answer questions such as does shared knowledge of the goal changes the best signals for predicting motion primitives? Does the addition of another follower, without knowledge of the goal, make it obviously or statistically more difficult for a leader to communicate intent? These and other related questions are answered in this work.

### 2.5 Similar human-human studies

Several human-human studies have been performed in an effort to better understand and characterize how people work together to accomplish basic tasks (Mojtahedi et al., 2017; Schmidts et al., 2016; Melendez-Calderon et al., 2015; Peternel et al., 2016; Bakar et al., 2007; Reed and Peshkin, 2008). Several others perform experiments that match the definition of co-manipulation used in this paper, using at least two people to move a large bulky object (Freeman et al., 2024; Maroger et al., 2022; Bin Abu Bakar et al., 2006; Mielke et al., 2017; Jensen et al., 2021; Ikeura and Inooka, 1995; Lanini et al., 2017). None of these studies were designed to explore the four fundamental aspects of co-manipulation explored in this work, and none of them incorporated teams of three at a time.

## 3 Methods

The human-human study that we describe in this section had the objective of enabling us to explore patterns of human dyad and triad behaviors in terms of motion and haptic signals during the co-manipulation of objects with significant size and mass. Using the resulting data from the study, we explore the following four areas: first, defining a static or rest state; second, identifying the best signal to use to predict predefined motion primitives corresponding to object degrees of freedom; third, the communicability of DOFs via haptics; fourth, paths used by participants to accomplish multi DOF tasks. This methods section gives a high-level description of the hardware [which is largely the same as was used in Freeman et al. (2024) except for a novel virtual reality interface] and then details the experiments performed to explore these four areas.

**Figure 1:**
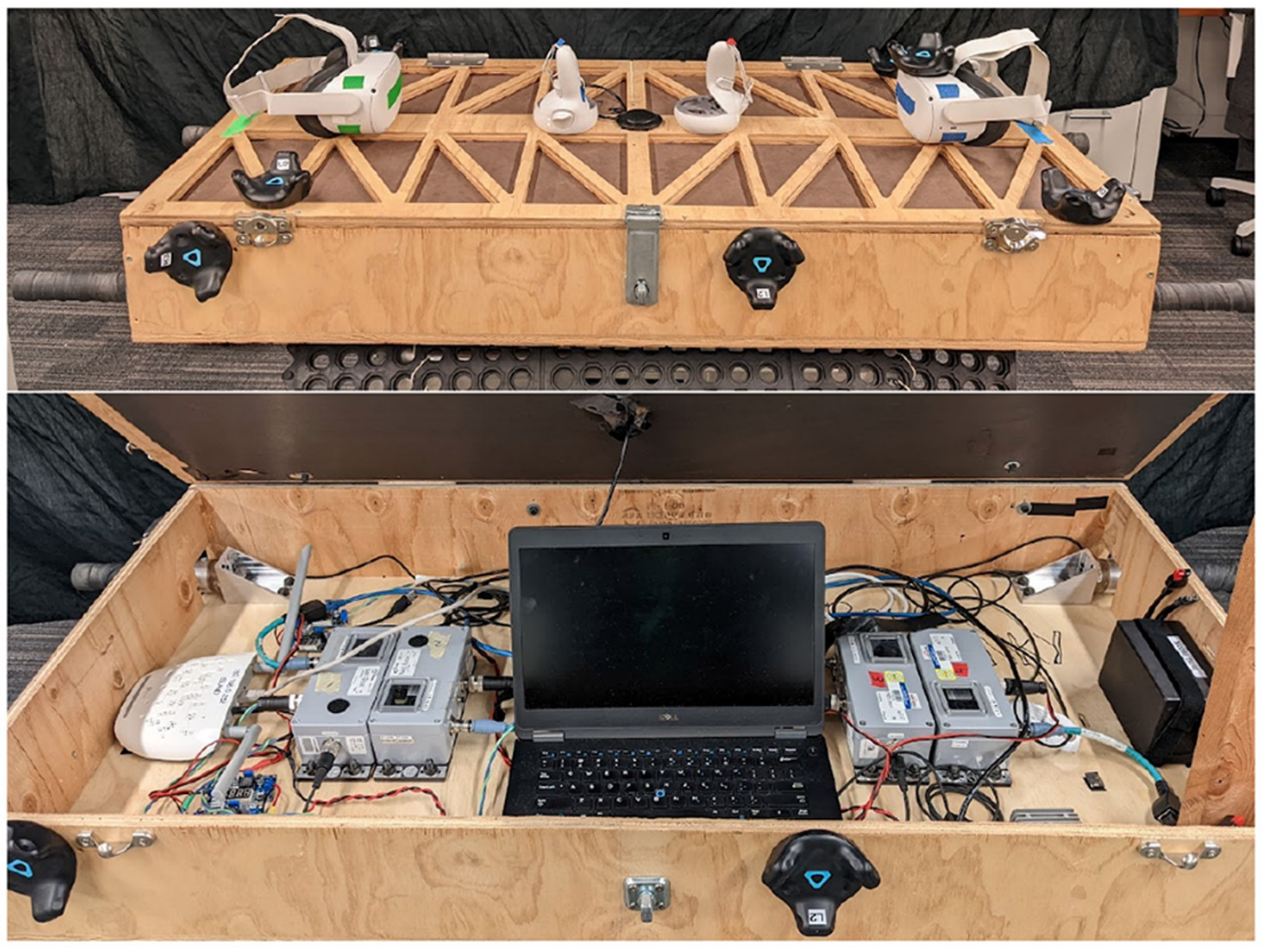
An external view of the table **(top)** showing black Vive Trackers and Oculus Quest 2 virtual reality (VR) headsets. An internal view of the table showing force-torque sensors and signal conditioning boxes **(bottom)**. This table design was modified and adapted from Freeman et al. (2024).

The table, or co-manipulated object, pictured in Figure 1, consisted of four ATI Mini45 force/torque (FT) Sensors and six HTC Vive trackers v2.0. These trackers were used for localization in a virtual reality (VR) environment that is described below and for collecting pose data of the table. We included two Oculus controllers for the follower(s) to visualize the location of the physical table accurately in VR, and a microphone to help ensure the data between multiple systems (VR, force, and pose data) was synced. We collected data (i.e. force, torque, position, velocity) at 200 Hz. The table is 1.3 m long by 0.5 m wide, weighing 25.3 kg and the table is the same that was used in Freeman et al. (2024). The justification for using four independent force-torque sensors (one on each handle) was that the goal of this study was to obtain ground-truth data when multi-agent teams manipulated large and heavy objects. Although this type of data would not be available to robots working in a multi-agent team, understanding the necessary signals for high performing teams requires that we initially obtain ground truth. Additionally, the reason for using four distinct handles instead of letting participants handle the object directly was to make sure that we accurately measured all forces applied by each participant. This obviously does not match manipulation of large, real-world objects like a couch or a fridge, but does allow us to understand the role of multiple agents and the forces they apply to heavy, bulky objects.

**Figure 2:**
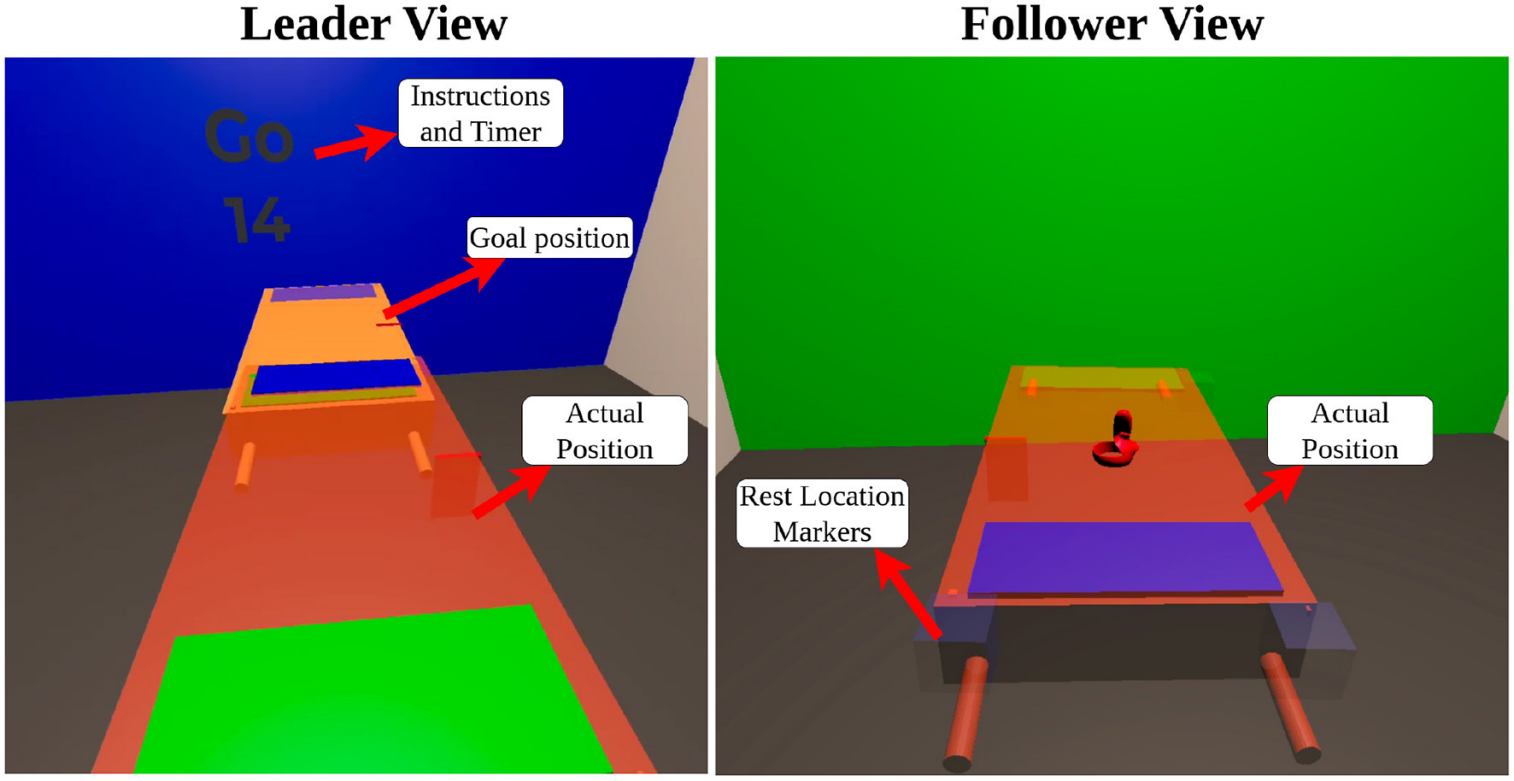
Leader and follower perspectives in VR showing the actual position of the table and the superimposed goal **(left)** and rest **(right)** locations.

Pose data from four Vive trackers were transformed to the geometric center of the table, and their positions were combined as a weighted average in the same manner as Freeman et al. (2024). The recorded path of the co-manipulated object was then smoothed and filtered, as described in detail in Appendix 2. This data was used to calculate velocity and acceleration, which were then transformed into the table frame. The table frame is located at the center of the co-manipulated object with the x-axis pointing toward the side with the blue rectangle as shown in Figure 2. The FT data was treated in the same manner as Freeman et al. (2024), yielding two force measures one for the leader and another for the follower(s). These two forces and torques were considered from the center of mass. An important distinction between the experiments of Freeman et al. (2024) and the current research is that although the object (i.e. table) was instrumented in a similar way, the tasks and co-manipulation maneuvers were very different. In Freeman et al. (2024), human dyads would move the table around five physical obstacles under different modi or descriptors of how to move (e.g. quickly, carefully). In the current research, the tasks involved shorter direct combinations of the 6 DOFs where one or more human partners were restricted in their knowledge of the goal (through a virtual reality environment).

We developed a virtual reality (VR) environment, which enabled us to carefully control visual information and cues given to each participant through Meta Oculus Quest 2 headsets. In the leader's headset, two copies of the table were displayed; one to represent and track the pose of the physical object and another to represent the goal pose (position and orientation), as shown in Figure 2. Written instructions were also provided on each of the four virtual walls stating when to start a task, time remaining, and when to return to the starting position. The followers' headsets had only an image of the table representing the real object and markers to help them return to the starting location before each task. These components are pictured on the right in Figure 2. Participants were provided a large enough area to feel confident moving in VR.

Importantly, participants were only allowed to communicate haptically throughout the study. This means that the only information available to each team member (both for dyadic and triadic trials) was the real-world position of the co-manipulated object (as rendered in VR and based on their own kinesthetic feedback), and the forces felt through the handles they were holding. We fully expect that allowing teams to share and communicate information through additional channels (such as visual or auditory information) would improve performance, especially in cases where the follower does not know the goal. However, from prior experiments (see Mielke et al., 2017, 2024) we have noted that humans are particularly adept at using only haptic information to successfully complete complex tasks. The goal in limiting the participants information to haptic feedback only is to observe the role of haptic signals in dyadic and triadic interactions, as well as providing a baseline for future development of robot teammates that will initially rely on only haptic feedback to successfully and collaboratively achieve a task.

**Figure 3:**
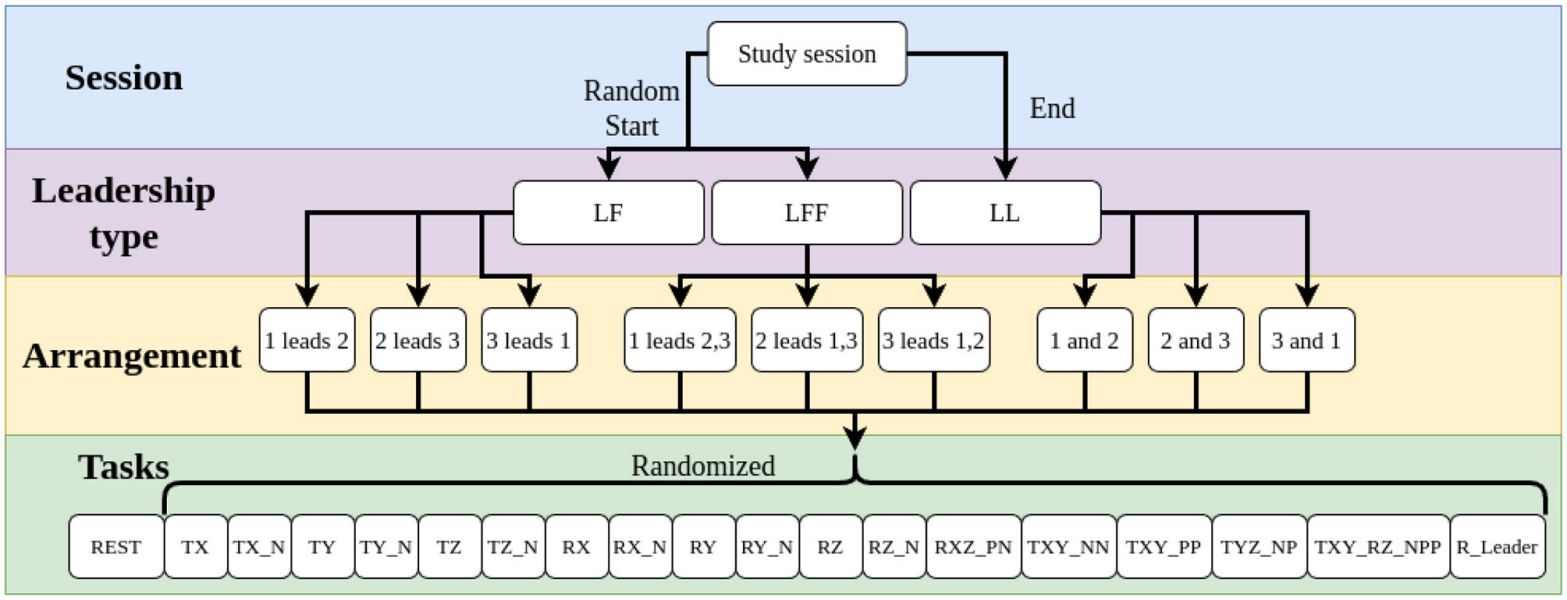
A flow diagram showing the typical execution order of each study session, with three different leadership types in nine different arrangements, resulting in 171 tasks per session. Explanations of the abbreviations for task names are detailed in Appendix 1.

Our study consisted of 16 sessions with 16 different teams. Each session, as represented in Figure 3, consisted of three volunteers. These volunteers watched a short instructional video, passed a fitness screening by lifting a 30 lb weight to shoulder height, and were instructed to move quickly throughout the experiment in order to complete all tasks in a reasonable time. They were also told to refrain from any verbal communication. The participants were randomly assigned a number, either one, two, or three. The group was then randomly assigned to start with either a leader and follower (LF), or one leader with two followers (LFF). While the leader-leader group type (LL) occurred last. The LL type was performed last because we expected the shared task objective information would impact learning the least.

In each leadership type a leader was on the green or negative x-axis side of the table facing the center, using both handles. Followers were arranged on the blue side also facing the center.

In each leadership type, participants completed three iterations of the tasks, changing positions for each iteration such that each participant experienced each role. In each of the nine resulting arrangements, participants completed a set of 18 tasks as shown in Figure 3.

**Figure 4:**
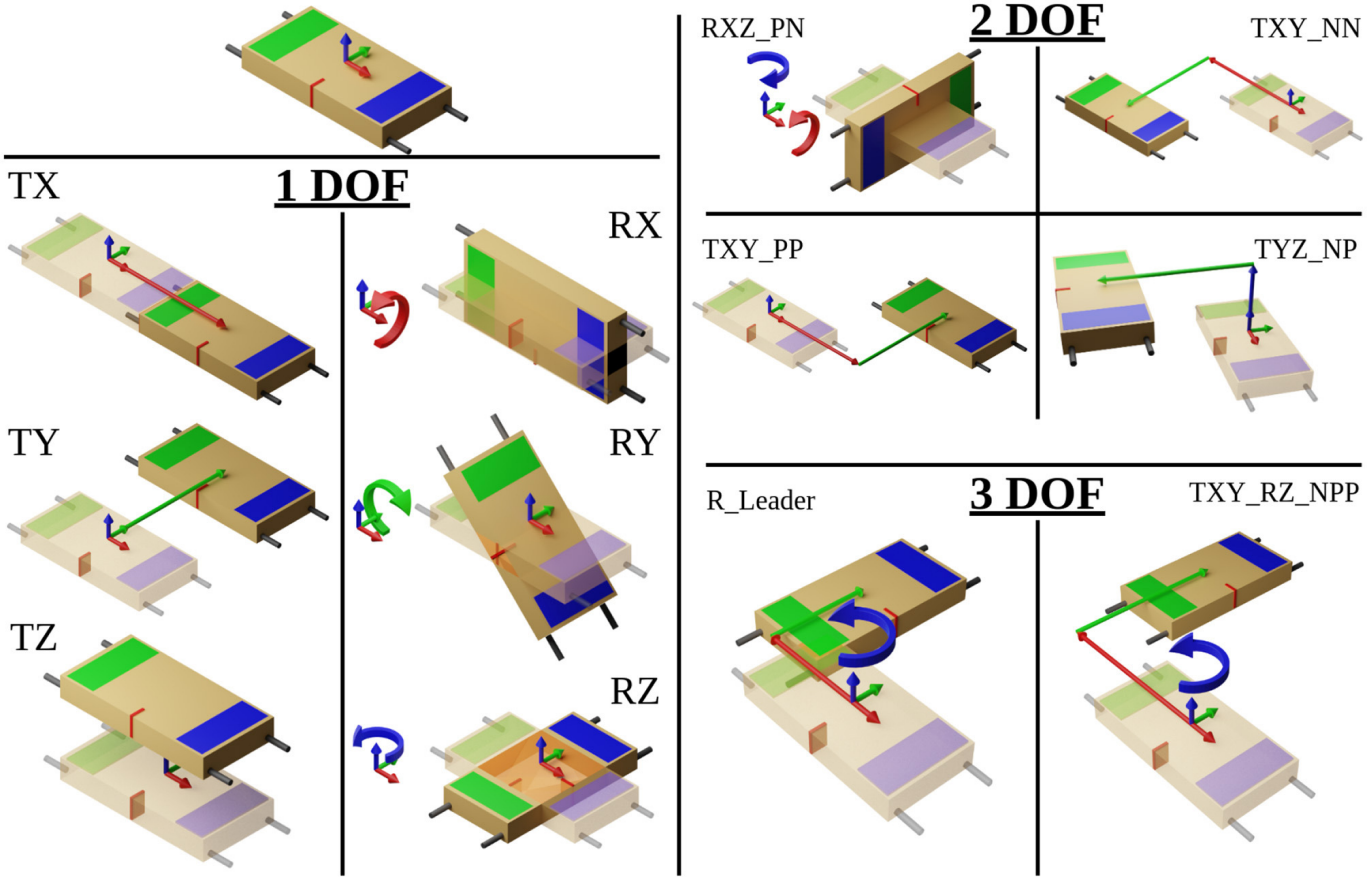
Visualizing Each DOF task, T stands for linear translation and R stands for rotation, the following letter(s) indicate the axis and direction. Further details are provided in Appendix 1.

Each set of 18 tasks contained twelve 1-DOF movements, one in the positive and negative directions for each Cartesian degree of freedom, four 2-DOF movements, and two 3-DOF movements. Six of the 1-DOF tasks and all the 2- and 3-DOF tasks are shown in Figure 4. The tasks were communicated to the leader(s), in a random order, via the Oculus headset. Participants were allotted 15 s to complete each task, which was shown to the leader(s) as a countdown timer on the virtual walls inside the environment.

After the completion of the task, either by arriving at the goal location and orientation or by running out of time, a period of 5 s was given for the participants to return to the starting location (which all participants could see). This process was repeated for the remaining leadership types, yielding 171 tasks per session. The LL set was omitted if a session had to be cut short, which occurred 3 times in the 16 study sessions. The following video links show a sample of one set of tasks from both VR and real-world perspectives: VR perspective, Real world perspective.

## 4 Results and analysis

We next summarize the main topics of interest as discussed in the following sections. First, we perform an analysis of the static, or at-rest, state, and identify the transition to an active state where the team is moving toward a new goal location. This is followed by an exploration of potential signals for predicting a desired motion primitive. Next, we address which DOF can and cannot be intuitively communicated via haptics, and a basic breakdown of the difficulty of communicating a desired motion in each DOF. Finally, we analyze four 2-DOF tasks to determine whether people tend to divide tasks up into separate DOFs, or if they combine them to take the most direct path.

### 4.1 Rest task analysis

The ability to differentiate between when to stop and when to move is one of the most basic and potentially most important co-manipulation skills. It is therefore crucial to be able to clearly differentiate between desired static and active states. For this reason, data was collected for participants standing at rest before the start of each of the nine leadership arrangements or combinations, as shown in Figure 3. For this analysis, we consider the following measures in both their linear and rotational forms: change in position, velocity, and acceleration. These measures were chosen because they must change in order to leave a static state. Our analysis will consider the levels of noise in each of these measures across different sessions or groups of people and across leadership types. The insights gleaned from the analysis on noise are used to propose and test a basic classifier for discriminating between static and active states. Note that data from one group was dropped due to intentional motion that we observed during their REST task.

**Figure 5:**
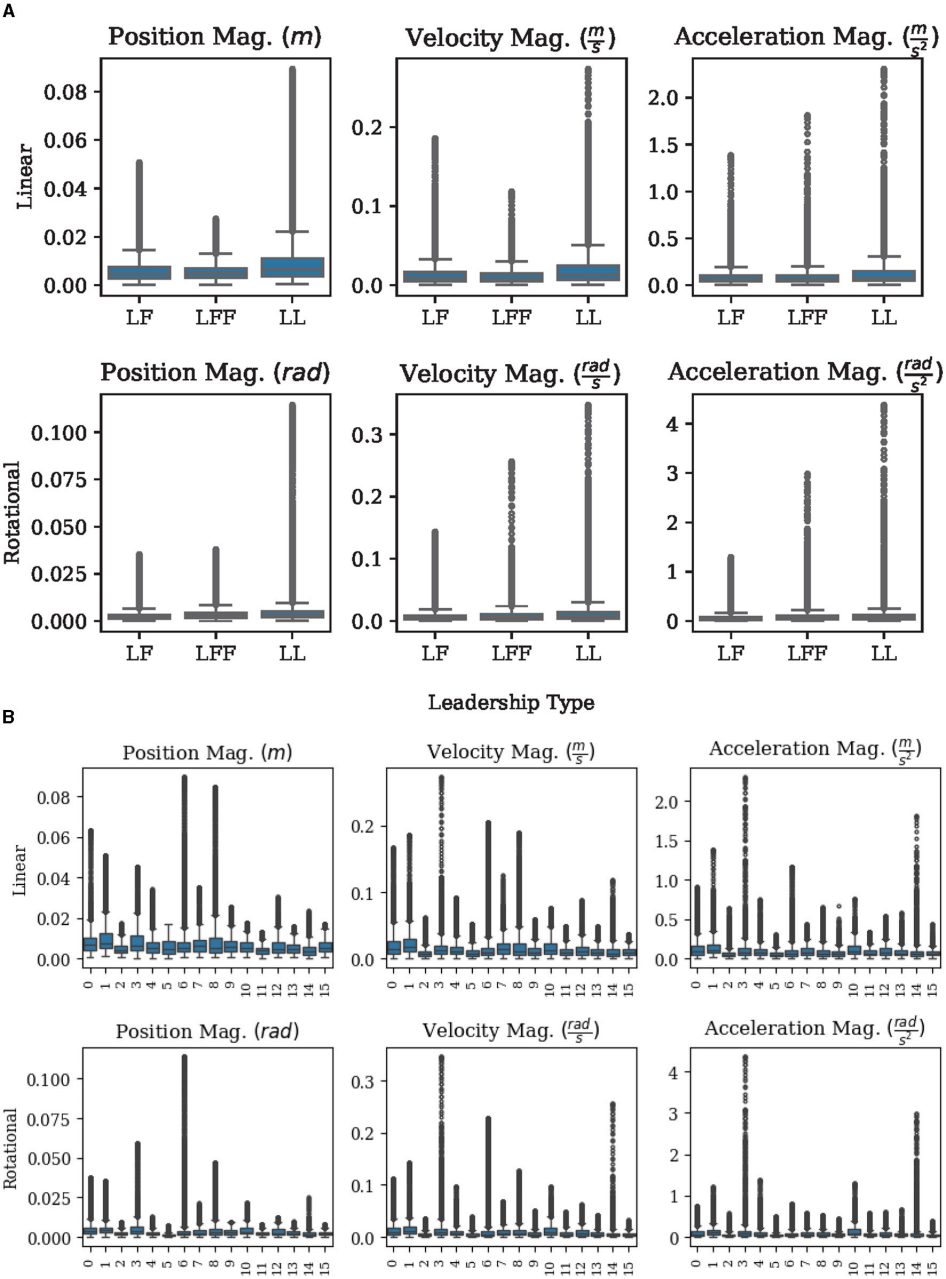
Standard box plots (showing the minimum, maximum, median, 25th and 75th percentiles, along with outliers) help show an exploration of measures. **(A)** A comparison of measures across different leadership types during REST tasks. **(B)** A comparison of measures across different groups during REST tasks.

First, we consider whether each measure is consistent across sessions and leadership types. A measure that is consistent across different study sessions and leadership types during the rest task suggests that the chosen measures will be robust to different groups of people and different arrangements that might occur in similar situations. The strong overlap of the box plots in Figure 5 shows that the measures chosen are practically indistinguishable across leadership types and groups. A closer inspection of each axis is made in Table 1 where the means and standard deviations of the linear and rotational, positions, velocities, and accelerations are given along each axis of the table frame, as well as their respective magnitudes. From Table 1 we can again observe a significant increase in standard deviations as we go from position measures to velocity and acceleration measures. We can also observe that the standard deviations of our selected measures are often nearly as small as the sensors can reasonably detect. This small range in noise should be ideal for detecting the transition from a static to an active state.

**Table 1:** Mean and standard deviation (SD) of the REST task and of stationary or unperturbed sensors.

**Mean and variance (SD)**			
**Measurement type**	**Rest task mean**	**Rest task SD**	**Sensor SD**
Position magnitude *m*	0.007	0.007	0.0006
x position *m*	0.000	0.006	0.0029
y position *m*	0.000	0.006	0.0018
z position *m*	0.000	0.004	0.0024
Velocity magnitude $\frac{m}{s}$	0.015	0.018	0.0023
x velocity $\frac{m}{s}$	0.000	0.015	0.0029
y velocity $\frac{m}{s}$	-0.001	0.015	0.0018
z velocity $\frac{m}{s}$	0.000	0.010	0.0024
Acceleration magnitude $\frac{m}{s^{2}}$	0.097	0.104	0.0296
x acceleration $\frac{m}{s^{2}}$	0.000	0.081	0.0418
y acceleration $\frac{m}{s^{2}}$	0.000	0.073	0.0243
z acceleration $\frac{m}{s^{2}}$	0.000	0.091	0.0306
Rotation magnitude *rad*	0.004	0.006	0.0004
x rotation *rad*	0.000	0.003	0.0005
y rotation *rad*	0.000	0.002	0.0006
z rotation *rad*	0.000	0.006	0.0004
Rotational velocity magnitude $\frac{rad}{s}$	0.010	0.016	0.0020
x rotational velocity $\frac{rad}{s}$	0.000	0.013	0.0019
y rotational velocity $\frac{rad}{s}$	0.000	0.007	0.0026
z rotational velocity $\frac{rad}{s}$	0.000	0.012	0.0013
Rotational acceleration magnitude $\frac{rad}{s^{2}}$	0.093	0.139	0.0279
x rotational acceleration $\frac{rad}{s^{2}}$	0.000	0.140	0.0287
y rotational acceleration $\frac{rad}{s^{2}}$	0.000	0.063	0.0419
z rotational acceleration $\frac{rad}{s^{2}}$	0.000	0.065	0.0186

**Figure 6:**
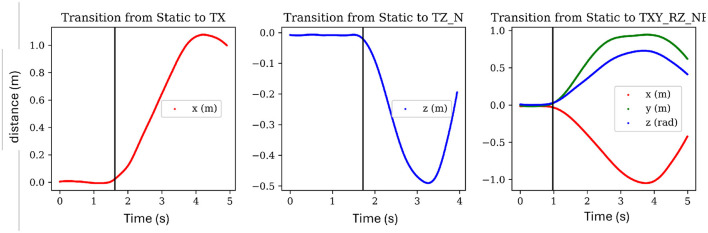
Showing the transition point between a static and active state for three randomly chosen tasks. The black line shows the estimated point of transition from a static state to an active state via the proposed method. Time is presented in seconds.

We reasoned that a simultaneous three-sigma (3σ) change in position and velocity along a particular axis would provide a robust trigger for identifying the transition from rest to an active state. This was chosen because a 3σ change in linear position of two centimeters would require a 3σ change in velocity of 0.054 $\frac{m}{s}$ for approximately 0.38 s. This puts our transition time (from static to active state) on par with the reaction time of the human foot reported by Pfister et al. (2014) to be 0.328 ± 0.048 s. Applying the 3σ bound to position is advantageous because it allows the object to act as a natural filter that handles the fluctuations and noise present in both velocity and acceleration. The 3σ bound on velocity is also robust because a 3σ rate in acceleration would have to be maintained for more than 5.7 s in order to reach a 3σ velocity. This approach of applying a simple 3σ threshold to both changes in position and velocity was applied to each axis for both rotation and translation using the REST task standard deviation values as reported in Table 1. In order to confirm that this deviation is reasonable based on the various sources of noise, the last column is added which presents the sensor variance (also as a standard deviation) for each signal and corresponding direction as labeled in the first column. A sample of the results of this transition method is presented in Figure 6, where plots of position are shown for three different tasks with a black line denotes the predicted change from a static to an active state. This method passes visual inspection in most cases and so is sufficient for this work. Future works should test the method in human-robot trials and can likely fine-tune the response by including other signals, considering different levels of sigma for each axis, or exploring less simplistic approaches.

### 4.2 Motion primitive signal analysis

As discussed in the prior works section, motion primitives have been used to improve the performance of robot in a variety of tasks, including co-manipulation. However, as was noted by Bussy et al. (2012), greater performance can be obtained by increasing the number of motion primitives, though that comes at the cost of making them harder to detect. We chose to define a motion primitive for each of the six degrees of freedom of the co-manipulated object, in order to generalize to all possible motions. To ensure the highest confidence when identifying those motion primitives, we decided to determine a hierarchy of signal reliability by exploring several possible signals. The first signal was velocity, as it is closest in relation to changes in position. Next was acceleration, because it is a precursor to velocity. The third potential signal was force because forces cause acceleration and can be measured directly with relatively low noise. Force, however, has significant complicating factors that prevent it from being straightforward to use. In co-manipulation, forces applied by each agent that do not perfectly align can cancel out components of other forces acting on the same system. These are commonly known as interaction, internal, or compensating forces (Donner et al., 2018; Shergill et al., 2003; Groten et al., 2009). These forces act on the system but do not contribute to the acceleration of the system. To address this complication, we will consider several different measures of force: first, the raw force measured on the follower's side in the axis of desired motion; second, the forces that contribute to the net force referred to as parallel force or (*f*_∥_), and contributes to the magnitude of velocity; and third, forces measured from the follower's side that are perpendicular (*f*_⊥_) to the net force of the system and contributes to the direction of the net force. A fourth but rather different measure of force considered is the force of tension or compression. We observed that participants in our study preferred to keep the co-manipulated object in tension as opposed to compression and therefore this measure is also tracked in case fluctuations in this measure might carry notable signals. These chosen measures of force are explored in more depth in Shaw et al. (2024).

**Figure 7:**
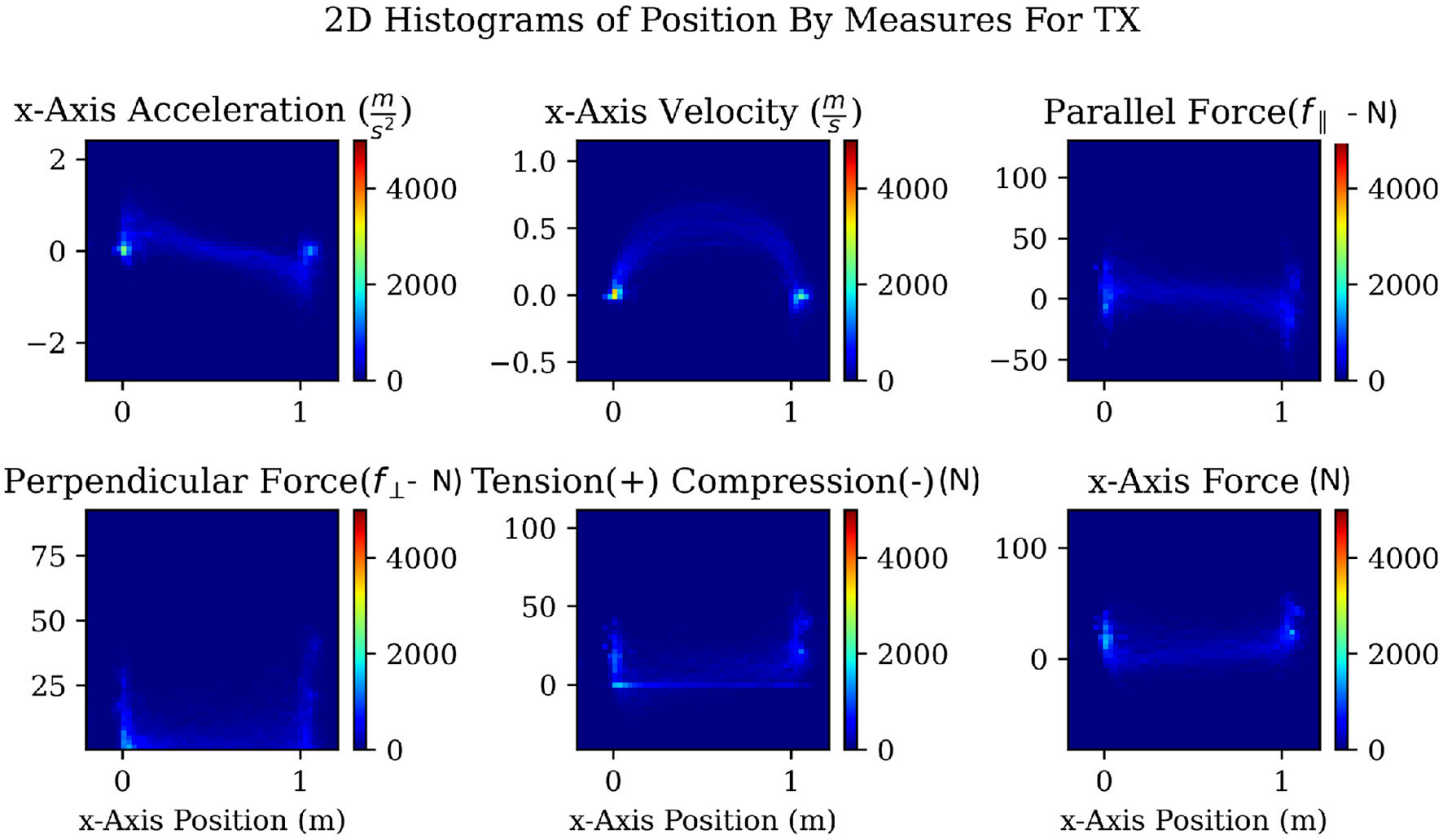
A 2-dimensional histogram for a task with a translation in the x-axis showing patterns over the change in position for acceleration, velocity, *f*_∥_, *f*_⊥_, tension and compression, as well as the raw measured force along the axis of movement. These plots were made and examined for each 1-DOF task. Only one set is shown here for space considerations. The colorbar is dimensionless and represents the count frequency in the respective cells of the figure.

To explore the chosen signals in search of patterns that might be exploited for the use of predicting our chosen motion primitives, we used two-dimensional (2D) histograms with the value of each given signal on the y-axis, and the change in position on the x-axis. Finally, the color indicates the frequency of a particular combination both a value of a measure at a particular position. Because we controlled for the position by setting the starting and ending locations for each task, any regularly repeating patterns show up as brighter paths on the 2D histograms, as can be seen in Figure 7. The 2D histograms in Figure 7 use data from all repetitions of the TX task, translating 1 meter in the positive x-axis as shown in Figure 4. We can observe several patterns present in the TX task. Accelerations follow a fairly consistent pattern that looks like a sideways “Z.” Velocities follow a smooth arc from the start to the end of the task. Tension, compression, and *f*_⊥_ forces drop from regions of high noise near the start and end of the task to low noise through the middle of the task. The measured force of the follower along the x-axis roughly follows the same pattern as acceleration. Finally, *f*_∥_ shows a pattern similar to *f*_⊥_ but not as sharp.

The 2D histograms sampled in Figure 7 enabled the visualization of patterns within the data and may also be used to effectively explore the data for other promising signals and combinations in future work. Because our goal was to calculate the correlation between each signal and the change in position along each axis, these 2D histograms helped to guide the type of correlations that we used. From the 2D histograms, we observed that most relationships were going to be nonlinear therefore a nonlinear correlation metric was needed. Distance correlation (Ramos-Carreño and Torrecilla, 2023) was chosen because it is the simplest correlation metric that handles nonlinear relationships. It works by examining how close sets of points are to each other and the spread or variation within each set of points. Our goal was to find the signal that would predict a motion primitive as quickly as possible. Therefore, whichever signal has the strongest correlation with the value we are trying to predict will be the best signal to track.

**Figure 8:**
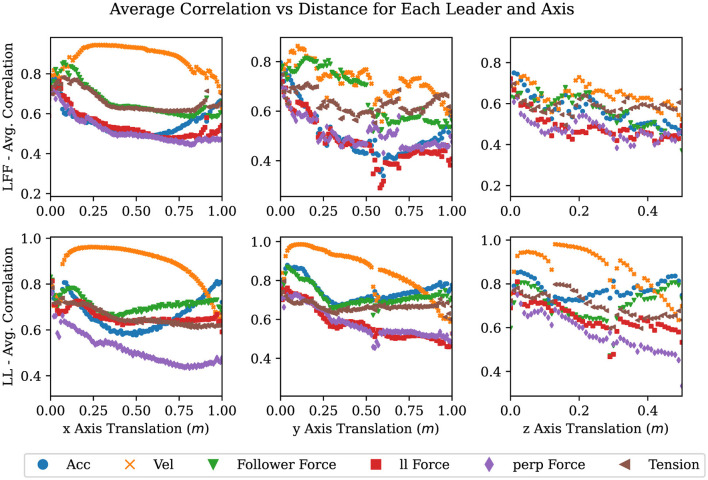
Plots showing averaged correlation values over changes in position where each column is a 1-DOF translation task in the + direction, and each row is a leadership type: LFF and LL.

**Figure 9:**
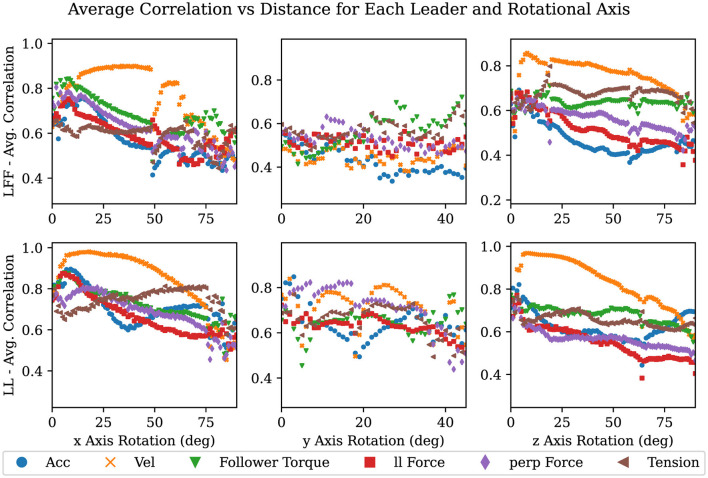
Plots showing averaged correlation values over the change in orientation where each column is a 1-DOF rotational task in both the + direction, and each row is divided by LFF and LL.

We proceeded by calculating the distance correlation with a growing window. The growing window approach allows us to track how the correlation coefficient changes over time and/or space. To apply this method, the distance correlation was repeatedly calculated starting with the first two points of a signal and location in each task and ending with all location and signal pairs collected for the duration of that task, giving us correlations over time. Then, we binned these correlations by 1-cm or 1-degree increments (in the case of rotations) in the direction of the goal and averaged them to obtain the average correlation up to that point in space for each task, as described in Algorithm 1. Finally, by averaging the distance correlation values within each bin across all the repetitions of each 1-DOF task (e.g. translation in and rotation about the x, y, and z-axes) within each leadership arrangement, we generated Figures 8, 9. Importantly, the trends between LFF and LF groups were very similar. We therefore only report the results for the LFF (as this type of group has not been extensively studied in the literature) and the LL group for comparison. Additionally, we only show this correlation for tasks moving in the positive direction as the data was symmetric and this improves the clarity of the accompanying plots.

**Algorithm 1:**
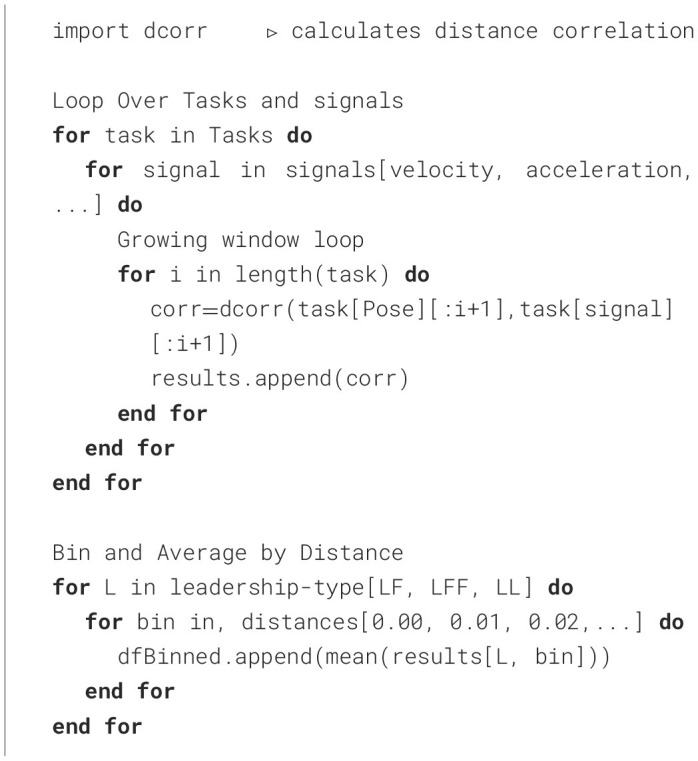
Calculate growing window distance correlation, from time series data of positions and signals, then bins and averages correlations into 1-cm bins.

In Figures 8, 9, the average correlation values of each signal are shown. Signals with high correlations, after minimal movement, close to zero on the x-axis, are likely to be superior predictors of a desired motion primitive. There are nine plots in each figure, one row for each leadership type and one column for each DOF. Each plot contains a scatter plot of the averaged correlation over the linear distance traveled along an axis in Figure 8, and the angular displacement in Figure 9.

As can be seen in nearly all of the plots in Figures 8, 9, velocity in a given axis is both the most strongly correlated and most consistent signal with respect to a change in position in the same axis. The exceptions to this observation are rotations about and translations in the y-axis. Rotations about the y-axis show no best signal to track suggesting that participants are inconsistent in how they perform or attempt this task. Translations in the y-axis show that for the LF and LFF groups a force on the followers side competes with velocity for the strongest correlation with the desired motion. This is likely due to torques from the leader creating a force on the followers side in the desired direction. Quantifying how and when velocity is strongly correlated with future motion and when it is not lends support to the observations of several researchers (Bin Abu Bakar et al., 2006; Mielke et al., 2017; Ikeura and Inooka, 1995; Cremer et al., 2014) who indicate velocity as an important signal to track for predicting future motion. It also sheds light on when velocity is the best signal and where it falls short as will be discussed later in this section.

Across leadership types, we can observe that in every case the LL type has the smoothest and most distinct correlation between change in velocity and change in position or orientation. This suggests that the noise or variance in these plots may correlate with the difficulty of communicating a particular motion primitive. We expect that this is because communicating a desired motion primitive would be more difficult for LF and LFF groups than for an LL group. This idea is further supported when we note how the noise increases from a translation in the x-axis to a translation in the y-axis where LF and LFF must overcome the translation vs rotation problem (Dumora et al., 2012). We also observed that the changes in noise correlated with the ability of the leader to produce a velocity in or about the desired axis on the follower's side. In other words, if the leader can produce the desired acceleration of the co-manipulated object on the follower's side, without the assistance of the follower, then the signal will be more clear, reliable, and consistent. For example, in a translation or rotation in the x-axis, the leader experiences no mechanical disadvantage when producing a velocity in or about the x-axis. Similarly, when performing a translation in the y-axis, the leader must create both a force and a torque on the leader's side to create velocity in the y-axis. The additional torque is needed to counter what is naturally created by accelerating the leader's end of the table, thereby preventing a rotation in the z-axis. The table in this study provides a lever arm of 0.25 m between each hand to counteract the torque created with the 0.64 m lever arm between the leader and the center of mass of the table. Finally, the moment arms available when performing a translation in the vertical axis are the width of the participant's hands, approximately 0.05 m versus the moment arm to the center of mass of 0.64 m. Given the large mechanical disadvantage present, it is surprising that a vertical movement can be communicated at all. The leader likely depends on the follower to simply guess the right direction and magnitude when vertical movements versus rotations about the y-axis need to be communicated. As evidenced by the high completion rate in vertical movement tasks and low completion rates in rotations about the y-axis, which are discussed in the following section, people default to keeping co-manipulated objects level rather than anticipating rotations about the y-axis.

It is surprising to note that rotations about the y-axis show almost no sign of a consistent signal under any leadership type. Even in LL where both participants knew the task, there is no consistent correlation. This seems to suggest that rotation about the y-axis, at least in this experiment, is unfamiliar; most people have not yet found a consistent and comfortable way to complete the task, which leads to a more random approach. Thus, we cannot expect participants to handle rotations about the y-axis intuitively in the same way we do with the other degrees of freedom. This may also just be indicative that additional communication channels (such as visual or auditory signals) may be necessary for some tasks to clarify intent and coordination. In contrast, both the data and video analysis showed that human teams could quickly overcome any ambiguity in the task when rotation was about the x- or z-axes. Future work includes identifying small impulses or other haptic or motion-based signals at certain times such that a human-robot partnership can overcome this problem more effectively (at least in the x- and z-axes), eventually matching human-only team performance.

It should also be noted from Figures 8, 9 that the level of variance is quite high early in the task and increases again upon getting close to the goal in all tasks. Initially, the participants have either not yet begun to move or have not built a significant velocity. Then, when close to the goal, the leader or team tries to make adjustments that are precise and small in magnitude. In these situations, velocity does not have sufficient time to become dominant relative to the other signals. This suggests that velocity is likely only the best signal in movements of sufficiently large magnitude. For smaller movements and adjustments, it is not yet clear which signal or combination of signals will be most reliable.

### 4.3 Communicability of different degrees of freedom

One of the primary reasons we tested motions in each of the 6-DOF (positive and negative), was to address what can and cannot be intuitively communicated with haptic information in human-human co-manipulation. To show these results from our study, we reported the success rates for each task performed in Table 2.

**Table 2:** This table compares the difference in average time to completion and success rates across leadership types for the 1-DOF tasks.

**Task**	**% Success rate**			**Comparing average time to completion**			
	**LF**	**LFF**	**LL**	**LF-LL (s)**	***P*** **-value**	**LFF-LL (s)**	***P*** **-value**
TX	100	98	100	0.8	0.000^***^	1.4	0.000^***^
TX_N	100	100	100	1.7	0.004^**^	1.3	0.018^*^
RZ	100	96	100	1.4	0.000^***^	2.8	0.000^***^
TY_N	100	100	100	2.3	0.000^***^	2.9	0.000^***^
RZ_N	100	96	100	1.9	0.000^***^	3.3	0.000^***^
TY	98	100	100	2.8	0.000^***^	2.5	0.000^***^
TZ_N	96	96	100	2.8	0.000^***^	3.0	0.000^***^
RX_N	100	96	100	3.3	0.000^***^	3.7	0.000^***^
TZ	100	94	100	3.4	0.000^***^	3.7	0.000^***^
RX	96	100	100	3.4	0.000^***^	4.4	0.000^***^
RY	45	50	100	7.9	0.000^***^	6.8	0.000^***^
RY_N	34	38	100	9.1	0.000^***^	9.0	0.000^***^

The data are sorted from least to greatest average difference in time from the LL type. *p*-values are reported from paired *t*-tests Virtanen et al. (2020) comparing the means from LF and LFF to LL. ^*^p < 0.05, ^**^p < 0.01, and ^***^p < 0.001.

Success was defined as arriving at the goal location with 85% accuracy within 15 s. The percentage accuracy was calculated by dividing the progress toward the goal by the total distance to the goal. An accuracy threshold of 85% was chosen for two reasons: a percentage was used so that a specific threshold did not have to be specified for each axis, and the level of accuracy was chosen based on what felt natural in tests while developing the study. It is important to emphasize that this was selected before the studies were completed, and was done to focus our results and analyses on gross motion that would be most useful for human-robot teams collaborating to load or unload a truck, move a stretcher, remove rubble, or other similar tasks where fine manipulation may be less important for large portions of the task.

Table 2 reports completion percentages across each 1-DOF task and leadership type, which indicates whether the task could be communicated within the given time and communication constraints (i.e. visual cues from the motion of the table, and haptic cues from interactions through the table).

When considering the completion percentage, it is clear that rotations about the y-axis are by far the most challenging task to communicate. It is surprising to note that translations in the z-axis had high success rates. The translation vs rotation problem (Dumora et al., 2012) and our hypothesis predicted that there would be significant confusion between translations in the z-axis and rotations in the y-axis, but only rotations about the y-axis seem to have been affected enough to show up in our criteria for success. This seems to suggest that there is some default human behavior, such as assuming that the object should stay level, that prompts us to default to a vertical translation when there is an ambiguous choice, as opposed to a rotation about the y-axis. We also note that there is little if any significant difference in levels of success between leadership types. With the exception of rotations about the y-axis, participants achieved very similar levels of success in all tasks. This suggests that neither the restriction of verbal and visual cues (relative to the other agents in the team) nor the addition of a second follower rendered leaders unable to communicate the task. However, it is possible to obtain a better perspective of the differences between leadership types by examining their performance in metrics other than a boolean completion of the task.

**Figure 10:**
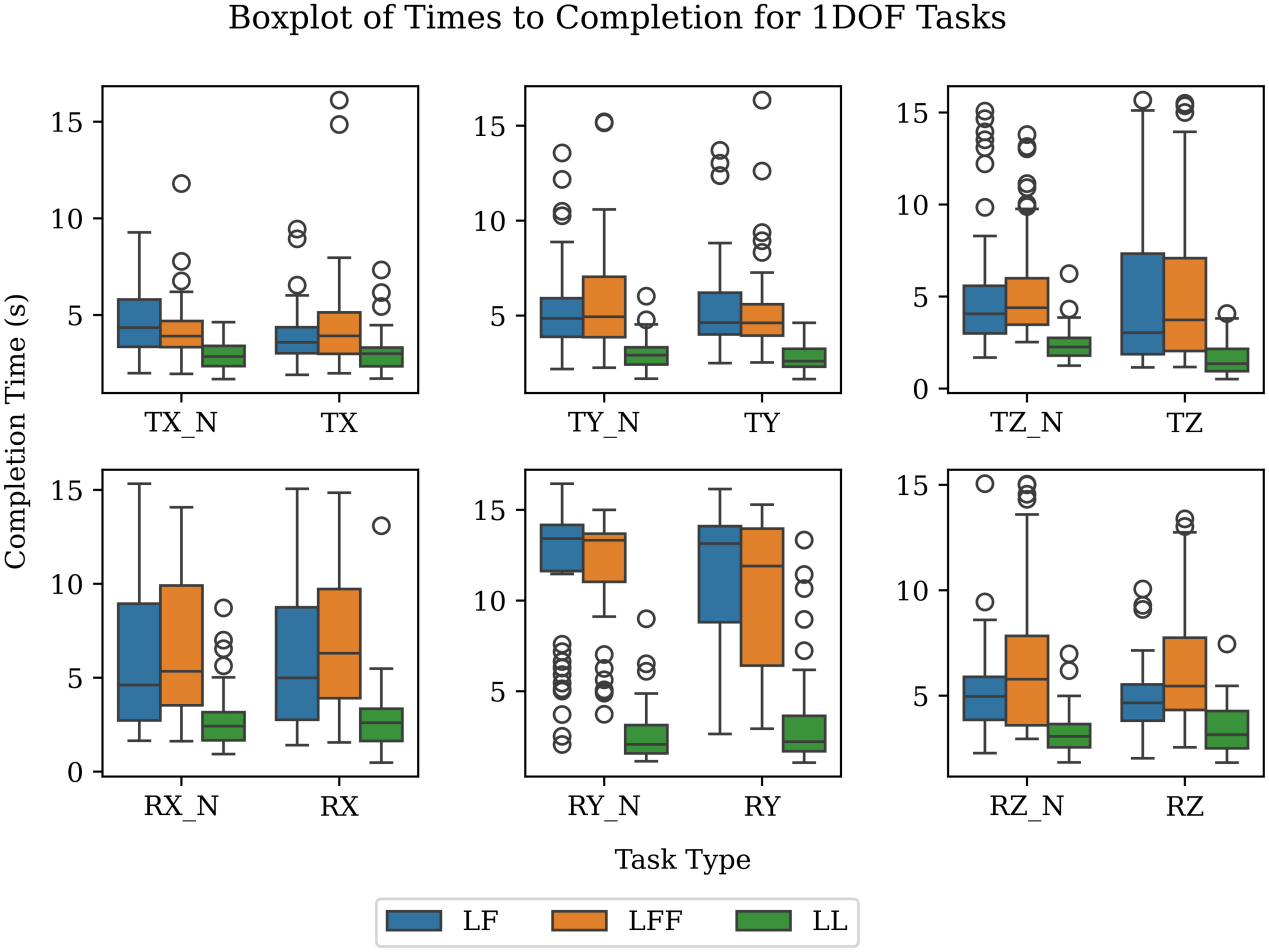
Average time to completion in seconds for 1-DOF tasks. The data are shown as box plots (with the minimum, maximum, median, 25th and 75th percentiles, along with outliers).

Since participants were instructed to complete the tasks quickly, the time taken to complete a task can be used as a simple performance metric. It is then possible to compare performance within specific tasks. Next, aggregating by leadership type allows us to correlate the average time to completion with the relative difficulty of completing a task. If all groups took, on average, longer to complete the task under a given leadership type, then we assume it was more challenging for them to complete. As discussed earlier, in the LL type both participants were simultaneously given the same instructions, (when to start, goal configuration, and time remaining), which resulted in near-ideal communication. Therefore, the average LL time can be used as a baseline performance against which to gauge the LF and LFF leadership types. When comparing against this baseline, we assume that longer average completion times are indicative of greater communication challenges. We can see in Figure 10 the significant jump in average time from LL type to LF and LFF types (see *p*-values in Table 2). This suggests that the increased difficulties in communication do indeed have a significant effect on the average time. We can also observe that there is little difference between the average completion times of LF and LFF types. This suggests the addition of a second follower does not cause a large difference in the communicability of the task. In our experiment, the addition of an extra participant was unlikely to enable groups to finish tasks significantly faster because in all cases there is only 6.3 kg per arm acting on the table. In addition, it may be that the just noticeable difference (JND) in forces is reduced for both followers in that each of them is responsible for manipulating a smaller percentage of the total mass of the object. Thus, the JND may be lower and allow the followers to respond earlier and possibly faster. Although adding a second follower might initially be considered a complication in the dynamics of the co-manipulation, we do not see a significant increase in time to completion. With more chances for disagreement to occur, it would not be surprising if a task with three participants took longer than with two but this was not evident in the experiments, or at least within the boundaries and context of this research.

By examining Table 2, we can form a rough idea of the hierarchy of DOFs from most to least difficult to communicate. Starting from the top, translations in the x-axis are the easiest followed by rotations about the z-axis and translations in the y-axis. These are followed by translations in the z-axis and rotations about the x-axis. The most difficult tasks to communicate are rotations about the y-axis.

### 4.4 Combining motion primitives

Having explored the signal space that can be used to predict motion primitives, as well as the communicability of motion primitives in each DOF, we now attempt to answer the following question: Given a task requiring two degrees of freedom (DOFs), do participants opt to handle one DOF at a time or attempt to accomplish them both simultaneously? To answer this question, we first needed to define a metric that would determine to what degree an individual task was completed by combining two DOFs versus splitting up the 2-DOF task into separate 1-DOF tasks.

**Figure 11:**
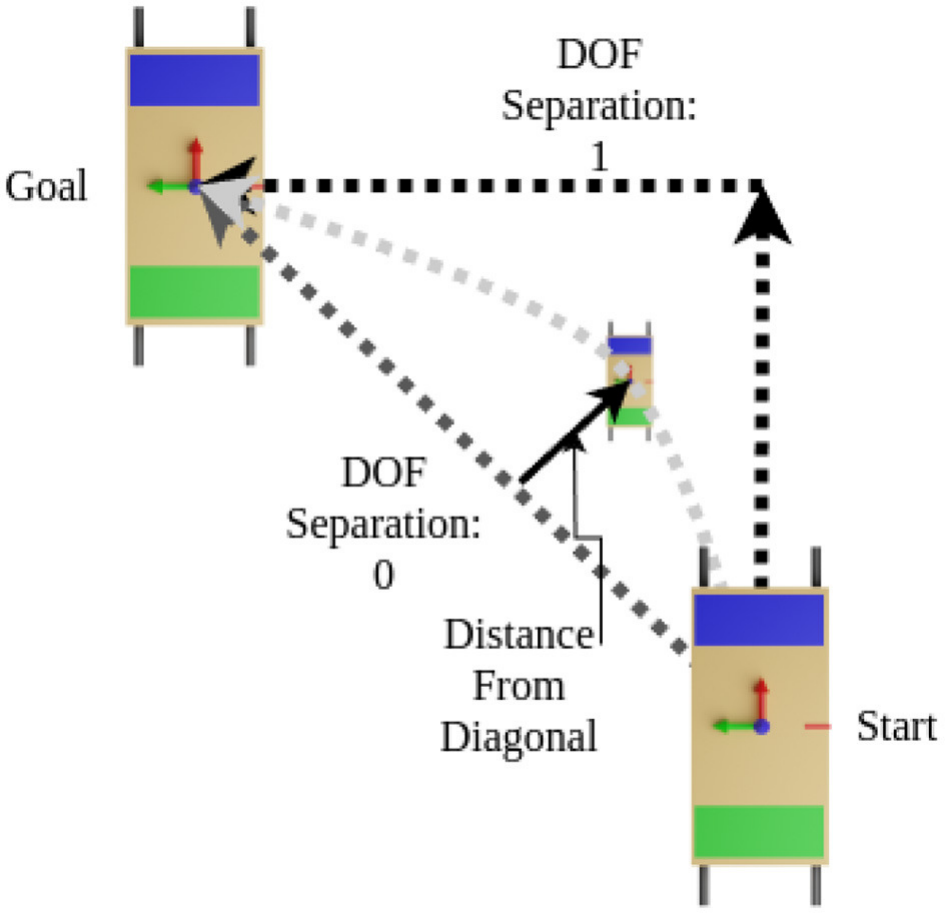
Explanation of how we developed our metric to quantify the separation of the degrees of freedom in a path. The diagram shows a path with perfect separation assuming a value of 1, a path with both DOFs used separately, and a hypothetical actual path.

To do this, we took the shortest path from a start point to a goal location, forming a right triangle with components along each axis. Following the shortest path would mean moving both DOFs simultaneously, while the right angle path would mean perfectly separating DOFs, as shown in Figure 11. To calculate the metric we use the pose of the table at each time step on the actual path taken by the participants, calculate its distance from the diagonal path, and normalize by the average distance between the right-angled path and the diagonal path. Finally, all of the normalized distances are averaged giving our DOF separation metric. A zero value indicates traveling in both DOFs simultaneously and a one indicates a perfect separation.

**Figure 12:**
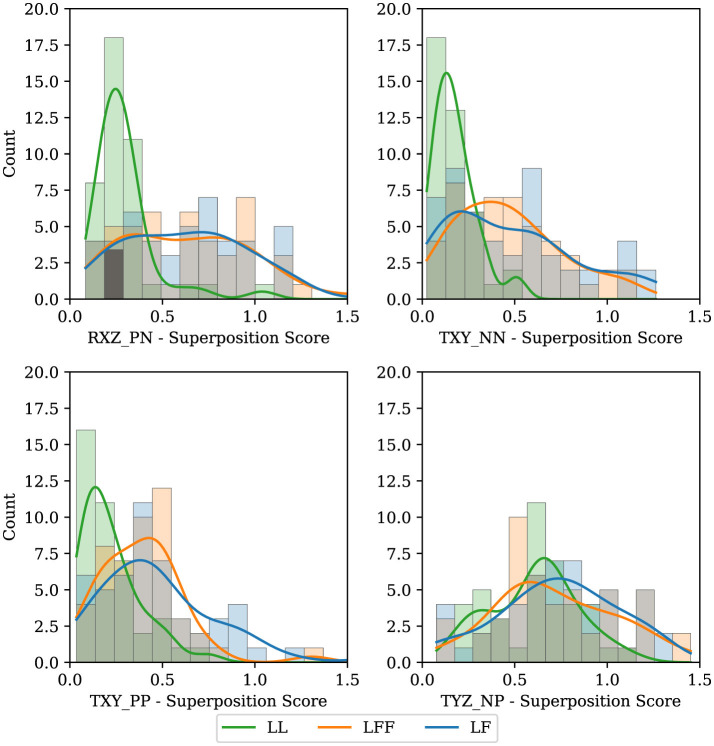
Histograms showing whether participants tend to combine DOFs to accomplish tasks or split them up. We compare across leadership types, LF (blue), LFF (orange), and LL (green).

By making a histogram of this multi-DOF metric across each of the four 2-DOF tasks and splitting by leadership type, we can obtain an understanding of how frequently and to what degree people combine or divide the DOFs. Where a zero indicates having taken the shortest path, a one indicates a separation of the degrees of freedom, and values greater than one indicate that a longer path was taken. This is shown in Figure 12. Consider tasks TXY_NN and TXY_PP (translation in the negative x and y directions, and translation in the positive x and y directions), which are more fully described in Appendix 1. Note that the histograms are skewed to the left, toward zero, especially for the LL type. This indicates that for planar tasks most people are unsurprisingly inclined to take the shortest path, and higher quality communication enables participants to be more direct. Next, observe the TYZ_NP task. In this task, participants move horizontally relative to the object being manipulated and then lift vertically. The histograms in this case show a natural splitting of the task into separate DOF movements, likely because it is more challenging to perform the vertical task before performing the horizontal movement. To perform the vertical task before the horizontal would require participants to hold the object in an uncomfortable position (uncomfortable in terms of human biomechanics) for a longer period of time. It is surprising that many participants opt to start lifting vertically before finishing the horizontal movement in the TYZ_NP task. Finally, in the RXZ_PN task, the LL configuration defaults to a very direct approach while the distribution of the LF and LFF types are nearly normally distributed, suggesting that there is a notable difference between this 2-DOF task and the others. Perhaps communicating two rotations simultaneously is more cumbersome than other 2-DOF tasks. With even more complicated maneuvers, such as a 3DOF task (e.g., rotation about two axes and translating in a third) or more, the analysis would necessarily require combining units (for distance and angle) in some way. Future efforts could explore how to take the aforementioned implementation of normalized trajectories and adjust them accordingly for higher DOF tasks with mixed units (i.e. meters and radians) or keep the various DOFs separate in a multi-objective sense.

## 5 Conclusion

Our work contributes several key components necessary for advancing natural robotic co-manipulation by addressing challenges present in the base-level motions, non-motion, and transitions in human co-manipulation. Our exploration of rest tasks identified primary signals involved in detecting a static state, as well as the subsequent transition into an active state. An exploration of the signal space clarified and validated that current velocity is a primary signal for determining future motion. It also revealed the limitations of velocity at low speeds, such as during the early stages of acceleration and fine positioning near the end of a task, which provided a clear area of study for future work. Our study also sheds light on what can and cannot be communicated intuitively via haptics, with the surprising result that, within our setup, all but one of the DOFs are communicable. Next was an analysis of how leadership types and different levels of information restriction affected the time-to-completion performance metric against a baseline level of performance, which was the average time to completion of the leader-leader teams. This baseline can be used in future human-robot co-manipulation implementations. Furthermore, an exploration was made into combining motion primitives, and a simple metric was proposed for measuring multi-DOF movement vs DOF separation to accomplish tasks. We showed that human co-manipulation path selection depends on goal location and communicability of the given task. Other works will also benefit from the simple and flexible nature of the study; any set or subset of the tasks can be replicated and compared against this human-human data.

The development of effective future co-manipulation controllers should take into account the following points discussed in this paper:

- By ensuring easy replication (full or partial), this study facilitates robust data comparison across future human-robot interaction research. This enables researchers to track performance metrics and behaviors, guiding the incremental development of robots and controllers.
- A three-standard deviation threshold on both position and velocity was shown to work well as a trigger, indicating a shift from a static to an active state.
- Velocity was shown to have the strongest correlation with changes in position in every DOF except rotations about the y-axis, but it is limited in its usefulness at the beginning and ends of tasks when velocity is low.
- The average times to completion for the LL type serve as a baseline performance against which future studies can be compared.
- The first-order ranking of communicability will allow future researchers to tailor their tasks to meet specific goals. For example, they might build a control algorithm to first tackle the easiest of tasks and then build on it to address more challenging tasks.
- Common approaches for human-human, two-DOF tasks can inform the development of more intuitive robotic controllers and serve as a reference point for evaluating behavior in diverse scenarios.

Future work in this area might involve quantifying levels of noise present within potential signals during multi-DOF movements and determining if velocity continues to be the best signal for determining future motion and whether it is suitable for identifying changes in direction. Likewise, future explorations could expand into more diverse types of co-manipulation, especially around the home, where furniture and appliances are repositioned, and at construction sites, where large and heavy boards, drywall, and piping are carried and manipulated by more than one person concurrently. In summary, this work improves our understanding of some of the most basic and fundamental behaviors in human co-manipulation, thus furthering the endeavor toward more effective and intuitive human-robot partnerships.

## Figures and Tables

**Algorithm 2:**
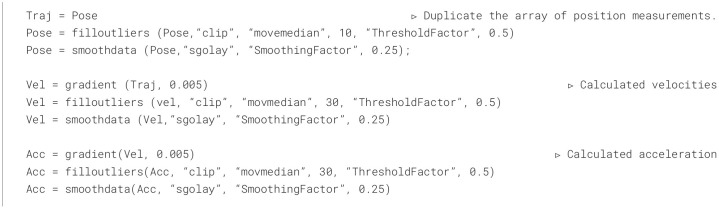
Outlier rejection smoothing and derivation.

## Data Availability

The raw data supporting the conclusions of this article will be made available by the authors, without reservation.

## Appendices

### 1 Nomenclature

**1 DOF**

RX: Rotation in x axis by 90 degrees
RX_N: Rotation in x axis by −90 degrees
RY: Rotation in y axis by 45 degrees
RY_N: Rotation in y axis by −45 degrees
RZ: Rotation in z axis by 90 degrees
RZ_N: Rotation in z axis by −90 degrees
TX: Translation in x axis by 1 meter
TX_N: Translation in x axis by −1 meter
TY: Translation in y axis by 1 meter
TY_N: Translation in y axis by −1 meter
TZ: Translation in z axis by 0.5 meter
TZ_N: Translation in z axis by −0.5 meter

**2 DOF**

RXZ_PN: Rotation in x axis by 90 degrees, and rotation in z axis by −90 degrees
TXY_NN: Translation in x axis by −1 meter, and translation in y axis by −1 meter
TXY_PP: Translation in x axis by 1 meter, and translation in y axis by 1 meter
TYZ_NP: Translation in y axis by −1 meter, and translation in z axis by 0.5 meter

**3 DOF**

R_leader: Rotation about the leader by 90 degrees about z via right hand rule
TXY_RZ_NPP: Translation in x axis by -1 meter, translation in y axis by 1 meter, and rotation in z axis by 90 degrees

### 2 Filtering and smoothing

The post processing of the position signals was performed in MATLAB with Algorithm 2 producing smooth and clean data streams for position (Pose), velocity (Vel), and acceleration (Acc) over time.

Algorithm 2 was visually tuned with a goal to capture both the quick jostling movements as well as the longer sustained movements as the table was moved to each pose, while rejecting measurements that would suggest improbable movements accelerations and velocity. This was accomplished by removing outliers and smoothing the data after each derivative calculation.
